# Investigating Lipid and Energy Dyshomeostasis Induced by Per- and Polyfluoroalkyl Substances (PFAS) Congeners in Mouse Model Using Systems Biology Approaches

**DOI:** 10.3390/metabo15080499

**Published:** 2025-07-24

**Authors:** Esraa Gabal, Marwah Azaizeh, Priyanka Baloni

**Affiliations:** 1 School of Health Sciences, Purdue University, West Lafayette, IN 47907, USA; egabal@purdue.edu (E.G.); mazaizeh@purdue.edu (M.A.); 2 Purdue Institute for Integrative Neuroscience, Purdue University, West Lafayette, IN 47097, USA; 3 Institute for a Sustainable Future, Purdue University, West Lafayette, IN 47907, USA; 4 Purdue University Interdisciplinary Life Science Program (PULSe), Purdue University, West Lafayette, IN 47907, USA

**Keywords:** energy metabolism, PFAS, PFESA-BP2, GenX, genome-scale metabolic models, PFOA, PPARα, lipid metabolism

## Abstract

**Background:** Exposure to per- and polyfluoroalkyl substances (PFAS, including 7H-Perfluoro-4-methyl-3,6-dioxaoctanesulfonic acid (PFESA-BP2), perfluorooctanoic acid (PFOA), and hexafluoropropylene oxide (GenX), has been associated with liver dysfunction. While previous research has characterized PFAS-induced hepatic lipid alterations, their downstream effects on energy metabolism remain unclear. This study investigates metabolic alterations in the liver following PFAS exposure to identify mechanisms leading to hepatoxicity. **Methods:** We analyzed RNA sequencing datasets of mouse liver tissues exposed to PFAS to identify metabolic pathways influenced by the chemical toxicant. We integrated the transcriptome data with a mouse genome-scale metabolic model to perform in silico flux analysis and investigated reactions and genes associated with lipid and energy metabolism. **Results:** PFESA-BP2 exposure caused dose- and sex-dependent changes, including upregulation of fatty acid metabolism, β-oxidation, and cholesterol biosynthesis. On the contrary, triglycerides, sphingolipids, and glycerophospholipids metabolism were suppressed. Simulations from the integrated genome-scale metabolic models confirmed increased flux for mevalonate and lanosterol metabolism, supporting potential cholesterol accumulation. GenX and PFOA triggered strong PPARα-dependent responses, especially in β-oxidation and lipolysis, which were attenuated in PPARα^−/−^ mice. Mitochondrial fatty acid transport and acylcarnitine turnover were also disrupted, suggesting impaired mitochondrial dysfunction. Additional PFAS effects included perturbations in the tricarboxylic acid (TCA) cycle, oxidative phosphorylation, and blood–brain barrier (BBB) function, pointing to broader systemic toxicity. **Conclusions:** Our findings highlight key metabolic signatures and suggest PFAS-mediated disruption of hepatic and possibly neurological functions. This study underscores the utility of genome-scale metabolic modeling as a powerful tool to interpret transcriptomic data and predict systemic metabolic outcomes of toxicant exposure.

## 1. Introduction

Per- and polyfluoroalkyl substances (PFAS) represent a class of synthetic chemicals widely used in various industrial products. Their structure consists of a hydrophobic fluorinated carbon backbone with hydrophilic ends. Different PFAS congeners have variance in characteristics depending on the alkyl-chain length [1]. Commonly termed as “Forever Chemicals”, PFAS are known for their low biodegradability, allowing their resistance in ecosystems. They favor the bioaccumulation in protein and lipid-rich organs such as the liver [2]. Therefore, hepatoxicity is among the primary effects associated with PFAS exposure, as extensively reported in rodent models [3,4,5,6,7,8]. Despite the lack of a definite mechanism of PFAS toxicity, research proposed that PFAS-driven hepatic dysfunction could happen through perturbations in lipid metabolism [9,10], hepatomegaly [11,12], elevation in peroxisomal β-oxidation, hepatocellular hypertrophy, and peroxisome proliferation [12,13,14]. Additionally, PFAS possess a surfactant nature, allowing them to potentially cross the blood–brain barrier (BBB). They are assumed to accumulate in the brain and cause neurotoxicity, which was demonstrated by the damage to endothelial cells and astrocytes in PFOS-exposed mice [15,16].

Previous in vivo studies of perfluorooctanoic acid (PFOA), a long-chain PFAS congener, showed lipid alterations in the liver via targeting Peroxisome Proliferator-Activated Receptor-α (PPARα), which acts as a transcriptional regulator of enzymes involved in lipid and fatty acid (FA) metabolism [17,18]. This nuclear receptor has long been considered a main molecular target for most PFAS congeners [19,20], and PFOA-driven transcriptional alterations in the mice liver were reported to be PPARα-dependent [21]. PFOA exposure has been reported to cause disturbances in amino acid, pyruvate, and TCA cycle metabolism, as well as glycerophospholipids and sphingolipid pathways [22]. Moreover, PFOA exposure in mice has been associated with AMPK signaling [23], an important modulator of energy metabolism, including glycolysis and the TCA cycle [24,25]. A study by Attema et al. showed high-dose PFOA exposure in mice is linked with liver transcriptomic changes related to fatty acid metabolism and oxidative phosphorylation [21]. GenX, a short-chain PFAS developed as a replacement for PFOA, was assumed to be less toxic due to its shorter half-life (3.375 days or ~81 h) [26]. However, in vitro and in vivo research showed its potent toxicity driven by perturbations in lipid and amino acid metabolism [10,27,28,29]. GenX exposure led to hepatic glycogen depletion in rat pups along with hyperglycemia, suggesting its role in developmental toxicity [11]. Moreover, liver from fetal rats showed gene expression changes associated with glucose metabolism and acetyl-CoA biosynthesis via *Pdp2* and *Pdk4* genes [11]. Similar to PFOA, GenX has also been associated with altered AMPK signaling [23]. While PFOA and GenX hepatotoxicity have been extensively investigated, PFESA-BP2 remains under-studied. Recent short-term PFESA-BP2 exposure studies in BALB/c mice [30] have shown dose-dependent regulation of liver carbon metabolism, including TCA cycle, fatty acid metabolism, and lipid accumulation, suggesting its potential role in disrupting liver metabolic homeostasis and causing hepatotoxicity [7,30].

While transcriptomic data provides a snapshot of gene-level regulation, further investigation is needed through the lens of metabolic networks, where the functional impact of gene expression changes on cellular metabolism can be more accurately addressed [31]. Given the complexity of biological systems, genome-scale metabolic models (GEMs) serve as powerful tools to dissect molecular changes. Through integrating multi-omics data with biochemical knowledge, GEMs enable a systems-level understanding of metabolic alterations that underpin molecular dysfunction associated with external exposure [32]. The use of GEMs was formerly reported to assist in predicting cancer-related therapeutic agents [33] and detecting metabolic variances across model organisms supporting translational research [34]. The human brain-region-specific GEM has enabled the detection of perturbed cholesterol, bile acid, as well as sphingolipid metabolism in Alzheimer’s Disease (AD), illustrating how metabolic changes contribute to disease progression [32,35]. In environmental toxicology, GEM application enabled optimization of growth conditions for *Paenarthrobacter aurescens*, a bacterium with bioremediation potential against atrazine, which is a widely used herbicide associated with environmental and health risks [36,37]. Moreover, GEMs of *Geobacter metallireducens* [38] and *Pseudomonas putida* [39] species have successfully modeled metal and pollutant degradation, enabling bioremediation insights by simulating electron acceptor dynamics and predicting xenobiotic metabolism pathways [36]. GEMs of *Nitrosomonas europaea* [40], *Geobacter sulfurreducens* [41], and *Rhodopseudomonas palustris* [42] have been applied to wastewater treatment, predicting pollutant degradation, ammonium removal, uranium reduction, and hydrogen gas production with high metabolic accuracy. These models offered a foundation for designing targeted genetic and bioremediation strategies across chemolithotrophic and photoheterotrophic systems [36]. Additionally, recent research from Pannala et al. [43] demonstrated hepatotoxic chemicals causing significant upregulation in metabolic genes and their corresponding pathways related to carbohydrate, amino acid, and lipid metabolism by integrating large-scale transcriptome data with the rat GEM. This approach successfully distinguished hepatotoxic from non-toxic chemicals and predicted associated changes in serum metabolite levels, identifying potential metabolic biomarkers of liver toxicity [43]. Central to these models is the gene–protein–reaction (GPR) rule, which maps annotated genes to their corresponding enzymes and the metabolic reactions they regulate. Consequently, alterations in gene expression can be systematically linked to changes in metabolic fluxes, enabling the prediction of functional shifts within the metabolic network [44]. In the present study, we coupled transcriptomics data with mice genome-scale metabolic reconstruction to generate context-specific models and predict the mechanism of hepatotoxicity associated with three PFAS congeners, PFESA-BP2, PFOA, and GenX. Our study highlights the use of systems biology approaches to identify metabolic alterations in response to PFAS exposure.

## 2. Materials and Methods

### 2.1. Acquisition of Liver Transcriptomics Datasets

We used the NCBI’s Gene Expression Omnibus (GEO) [45] database to extract total and bulk RNA sequencing datasets of mouse liver tissue exposed to PFAS. We chose three studies with the accession IDs: GSE147331, GSE147332, and GSE212294. In the first two studies, male and female BALB/c mice of 10–12 weeks of age were exposed to PFESA-BP2 (commercially known as Nafion byproduct-2) at five doses of 0.00, 0.03, 0.30, 3.0, and 6.0 mg/kg.bw. The mice were exposed for seven days (acute exposure) via oral gavage with ad libitum access to food and drinking water [30]. The third study consisted of male C57BL/6J wildtype and PPARα^−/−^ mice of 9–10 weeks of age exposed to two doses of PFOA (0.05 and 0.30 mg/kg.bw/day) and one dose of GenX (0.30 mg/kg.bw/day). The mode of exposure was through drinking water for 20 weeks (chronic exposure) [21]. The mice had ad libitum access to high-fat diet (45% kcal fat) and drinking water. In all studies, mice were euthanized following the end of exposure, and liver tissues were collected for performing total RNA sequencing. Transcriptomics data for these studies were deposited in GEO database.

### 2.2. Transcriptomics and Pathway-Based Enrichment Analysis of Metabolic Genes

We obtained the raw gene counts for GSE147331, GSE147332, and GSE212294 datasets and mapped them to the metabolic genes in mouse genome-scale metabolic model, iMM1865 [44]. Differential gene expression analysis was conducted via DEseq2 [46] package in R programming language (v.4.2.2) [47]. Genes with a log FC of 0.25 and adjusted *p*-value < 0.05 were considered as differentially expressed genes (DEGs). Pathway enrichment analysis was performed for DEGs using enrichr-KG [48], and we selected Reactome-2022 [49], GO-(Biological-Process) [50,51], MGI-(Mammalian-Phenotype) [52], and KEGG-Human [53] libraries for the analysis. The output of biological pathways and associated genes was visualized using R package of ggplot2 [54], while Venn diagrams were generated using packages ggVennDiagram [55] and VennDiagram [56]. Principle component analysis (PCA) of VST (Variance stabilizing transformation) transformed normalized gene count was conducted using plotPCA in DEseq2 package. Visualization in boxplots and violin plots was conducted through using tidyplots [57]. For easier illustration of differences between groups in GSE212294, we gave new identifications for the exposure groups: Group 1 (WT-Ctrl), Group 2 (KO-Ctrl), Group 3 (WT-GenX), Group 4 (KO-GenX), Group 5 (WT-low PFOA), Group 6 (KO-low PFOA), Group 7 (WT-high PFOA), and Group 8 (KO-high PFOA). Here, KO represents the PPARα^−/−^ genotype, while low-PFOA and high-PFOA reflect the two PFOA doses of 0.05 and 0.3 mg/kg, respectively.

### 2.3. Transcriptomics Data Integration with iMM1865 Mouse GEM

To investigate the physiological changes associated with PFAS congeners’ exposure, we integrated liver transcriptomics data with the mouse genome-scale metabolic model (GEM), iMM1865 [44]. The mouse GEM contains 1865 metabolic genes, 10,612 metabolic reactions, 93 subsystems, and 5839 metabolites [44]. For generating condition-specific metabolic models of mice, we implemented iMAT (Integrative Metabolic Analysis Tool) [58] algorithm using COBRA toolbox v3.0 [59] in MATLAB 2019a, with the academic license of Gurobi Optimizer v.12.0 to solve LP and MILP problems. This resulted in the generation of context-specific metabolic models for all samples considered for this study. We performed flux balance analysis (FBA) for all context-specific models by optimizing BIOMASS reaction as the objective function. Following the approach in Baloni et al. [31], we considered reactions with flux value < 1 × 10^−6^ as inactive (carrying no flux), and these were excluded from the analysis. The reactions belonging to same subsystems were grouped together by taking their average flux and subtracting it from control group to detect subsystem-level perturbations due to PFAS exposure [31]. In case of GSE212294 study, we considered the WT-control group as the comparison reference, and hence, we could detect the influence of knocking out PPARα on the liver metabolic profile along with its role in exposure effects. We used scale-invariant geometric data analysis (SIGDA) [60] method for projective decomposition of flux data to rescale the flux matrix so that both subsystems and exposure conditions are comparable in magnitude, without distorting relative flux patterns. This enabled unbiased clustering and visualization of metabolic subsystem perturbations [60].

Changes in metabolic reaction fluxes were investigated using flux variability analysis (FVA) [61]. Minimum and maximum flux values were computed for reactions within model subsystems involved in metabolism of lipids, fatty acids, cholesterol, bile acids, triglycerides, glycolysis, TCA, oxidative phosphorylation, and ATP production. Subsystems were classified as activated if the fluxes of their associated reactions were higher than those observed in the corresponding control groups (i.e., vehicle control for the PFESA-BP2 study or Group 1 for the PFOA and GenX study). Conversely, subsystems were considered suppressed if the reaction fluxes were lower than those of the respective controls. Flux sampling was performed to explore the entire solution space of feasible flux distributions that satisfy the metabolic model’s constraints, including mass balance, reaction bounds, and the objective function [62]. OptGPSampler algorithm was used to generate 1000 flux samples per reaction to evaluate the distribution and variability of possible metabolic states across mouse models. FBA, FVA, and flux sampling were carried out in COBRApy [63], in Python v.3.9.6 while using Gurobi Optimizer v.12.0 to solve LP and MILP problems.

### 2.4. Statistical Analysis

Differential gene expression analysis was performed using Wald test in DEseq2, followed by multiple hypothesis correction of *p*-values using Benjamini–Hochberg (BH) method [64]. Genes were considered significant if they passed the thresholds of adjusted *p*-value (FDR) < 0.05 and an absolute log2 fold change (|LFC|) > 0.25. Considering the control group as the comparison reference in reaction fluxes, statistical analyses were performed using unpaired two-tailed *t*-tests. A *p*-value < 0.05 was considered statistically significant. Statistical analyses were conducted in R programming language (v.4.2.2).

### 2.5. Data and Code Availability

Transcriptomic data with the accession numbers GSE147331, GSE147332, and GSE212294 were downloaded from GEO database. The normalized gene expression mapped to metabolic genes in iMM1865, the mouse genome-scale metabolic model, along with all analysis scripts, are publicly available on GitHub at (https://github.com/BaloniLab/PFAS_Congeners_Mouse_GEMs.git) (1 June 2025).

## 3. Results

### 3.1. PFESA-BP2 Hepatotoxicity in BALB/c Mice Targets Lipid Metabolism in a Sex- and Dose-Dependent Pattern

**Figure 1:**
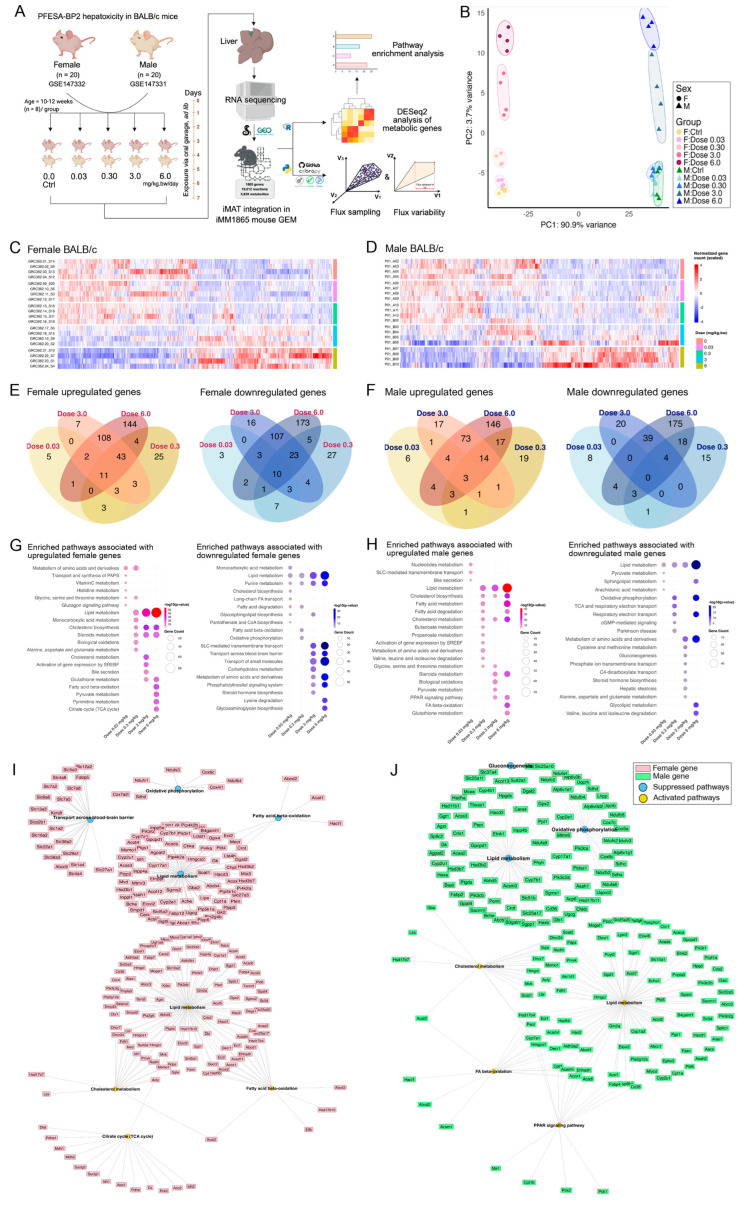
Hepatotoxicity of PFESA-BP2 in female and male BALB/c mice. (**A**) Experimental design of the exposure conditions in GSE147331 and GSE147332, with systems biology approaches applied in present study. (**B**) Principal component analysis (PCA) of normalized gene count across both female and male mice groups exposed to PFESA-BP2. Female and male are represented as F and M, respectively. (**C**,**D**) Heatmaps showing normalized and scaled gene count for differentially expressed genes (DEGs) in female mice (743 genes) and male mice (597 genes), across five doses of PFESA-BP2, respectively. (**E**,**F**) Venn diagrams representing DEGs in females and males, respectively, at all exposure doses. (**G**,**H**) Enriched biological pathways associated with the DEGs in female and male mice, respectively. The circle size represents number of genes corresponding to enriched pathway, and color represents statistical significance of enrichment (−log10 *p*-value). (**I**,**J**) Network analysis of lipid and energy metabolic pathways. Rectangle and circle nodes represent genes and pathways, respectively. Yellow circles are the pathways associated with upregulated genes, while blue circles are those associated with downregulated genes. Female and male networks are colored in pink and green, respectively.

PFESA-BP2 (commonly known as Nafion byproduct-2) is a long-chain PFAS, with a structure resembling PFOS (perfluorooctane sulfonate), and is reported to have a half-life of 296 days [65,66]. We used the mouse genome-scale metabolic model, iMM1865 [44], as a template model for this study and mapped metabolic genes to liver transcriptomics data from GSE147331 (male BALB/c) and GSE147332 (female BALB/c) exposed to PFESA-BP2 (Figure 1A) [30]. The mapped genes and expression values are provided in files uploaded on GitHub. Sex appeared to be a major factor for variance, as shown in the principal component analysis (PCA). PCA revealed distinct clustering of male and female along PC1, which explained 90.9% of the variance, while PC2 accounted for 3.7% of the variance and was primarily driven by the dose-dependent effects of PFESA-BP2 (Figure 1B). So, we focused on sex-specific metabolic changes due to PFESA-BP2 exposure, and the PCA of sex-specific analysis is provided in Supplementary file S2.

We used control group (0.0 mg/kg) as a reference for the DEG analysis in both datasets. In female mice, 359 upregulated and 384 downregulated genes were identified (Figure 1C; Supplementary file S1). The number of DEGs varied across doses: 0.03 mg/kg dose yielded the fewest DEGs (28 upregulated and 42 downregulated), followed by 0.3 mg/kg (103 up, 90 down), 3.0 mg/kg (182 up, 168 down), with the highest number observed at 6.0 mg/kg (330 upregulated and 336 downregulated) (Figure 1C). Since 29 genes exhibited varied expression across different doses, we focused on the ones that showed consistent up- or downregulation across all dose levels for the dose-comparison (Figure 1E). Similarly, male mice showed a total of 597 metabolic DEGs across doses (328 upregulated and 287 downregulated) (Figure 1D). Number of DEGs differed corresponding to the dose: 0.03 mg/kg dose got the least number of DEGs (26 up, 18 down), followed by dose 0.3 mg/kg (66 up, 49 down), dose 3.0 mg/kg (120 up and 67 down), and dose 6.0 mg/kg with (272 up, 251 down) DEGs (Figure 1F). For visualizing the dose-comparison, we excluded 18 genes with ambiguous expression across doses (Figure 1F). Together, these findings reveal a clear sex- and dose-dependent transcriptional response in metabolic genes, with higher doses (3.0 and 6.0 mg/kg) inducing the highest number of DEGs in both sexes, highlighting potential sex- and dose-specific molecular signatures.

We performed gene set enrichment analysis (GSEA) of metabolic DEGs identified across both sexes and four exposure doses to identify metabolic alterations due to PFESA-BP2 exposure. Lipid metabolism emerged as the most significantly enriched pathway linked to upregulated genes in both sexes across all doses. Interestingly, it was also the top-enriched pathway among downregulated genes in both sexes, except at the lowest dose (0.03 mg/kg) (Figure 1G,H). In both male and female mice, upregulated genes associated with lipid metabolism were mostly involved in fatty acid biosynthesis and elongation (lipogenesis), such as *Elovl1*, *Elovl6*, *Fasn*, *Fabp4*, *Acly*, and *Acss2* [67,68], along with cytochrome P450 enzymes of *Cyp1a2*, *Cyp2c55*, and *Cyp4a31* [69]. Other upregulated genes were involved in fatty acid β-oxidation, such as *Cpt2*, *Ehhadh*, *Abcd1*, *Hsd17b4*, and *Slc25a20* [70] (Figure 1I,J; Supplementary file S1). Downregulated genes associated with lipid metabolism in both sexes were involved in glycerophospholipid and triglycerides metabolism, which included *Agpat2*, *Dgat2*, *Gpcpd1*, *Crls1*, and *Enpp2.* Other downregulated genes belonged to sphingolipid and ceramide metabolism, including *Fabp5*, *Ugcg*, and *Asah1* (Figure 1I,J; Supplementary file S1).

Cholesterol biosynthesis and steroid metabolism were the second most significant pathways in enrichment associated with upregulated genes in both sexes, under all doses except the lowest dose (0.03 mg/kg) (Figure 1G,H). The genes included *Lss*, *Hmgcr*, *Sqle*, *Mvk*, *Hmgcs1*, and *Dhcr7*. The same set of genes, along with *Fdps*, *Elovl6*, *Fasn*, *Mvd*, and *Fdft1*, were involved in the activation of SREPFs (Sterol Regulatory Element-Binding Transcription Factors) signaling at a dose of 3.0 mg/kg in female mice and a dose of 0.3 mg/kg in male mice (Figure 1I,J; Supplementary file S1). Interestingly, *Cyp7a1* was upregulated in male mice while downregulated in female mice following the exposure (Figure 1I,J; Supplementary file S1). This gene encodes for cholesterol 7α-hydroxylase, which is the main enzyme in bile acid biosynthesis from cholesterol [71]. High doses of PFESA-BP2 (3.0 and 6.0 mg/kg) significantly activated PPAR signaling in male mice via upregulating the expression of *Fabp4*, *Acox1*, *Ehhadh*, *Me1*, *Pck1*, and *Cyp7a1*. Activation of PPAR signaling was not observed in female mice (Supplementary file S1). These results illustrate that PFESA-BP2 disrupts liver function primarily through modulation of key enzymes and signaling pathways related to lipid metabolism in a sex- and dose-dependent manner.

### 3.2. PFESA-BP2 Hepatotoxicity in BALB/c Mice Is Associated with Energy Dyshomeostasis

Lipid metabolism plays a critical role in fulfilling the body’s overall energy demands, with lipids serving as a major energy source alongside glucose [72]. We investigated the influence of PFESA-BP2 on energy metabolism, hypothesizing that such perturbations in hepatic lipids will result in energy dyshomeostasis. Female mice exposed to a high dose of PFESA-BP2 (6.0 mg/kg) exhibited significant enrichment in citric acid (TCA) cycle via upregulating the expression of *Aco2*, *Mdh1/2*, *Idh1/2*, and *Suclg1/2*. The same dose resulted in enrichment of pyruvate metabolism via upregulating the expression of genes including *Eno1*, *Pkm*, *Pdha1*, *Pdhb*, *Pck2*, and *Me1/2* (Figure 1I,J; Supplementary file S1). Fatty acid β-oxidation was enriched in female mice exposed to PFESA-BP2 doses of 6.0 mg/kg and 0.3 mg/kg, for both upregulated and downregulated genes (Figure 1G,I). Similarly, oxidative phosphorylation was enriched among pathways associated with downregulated genes in female mice exposed to the 0.3 mg/kg dose of PFESA-BP2. Interestingly, purine metabolism was also consistently enriched across all doses and similarly linked to downregulated gene expression, indicating broader perturbations in nucleotide and energy-related pathways (Figure 1G).

Our analysis indicated male mice had significant enrichment of pyruvate metabolism and β-oxidation associated with upregulated genes under exposure doses 3.0 and 6.0 mg/kg, respectively (Figure 1H). Pyruvate metabolic enrichment was modulated by increasing the cellular expression of *Aldh3a2*, *Css2*, *Me1*, *Akr1a1*, *Pck1*, *Dld*, and *Pck2* (Figure 1J). Both oxidative phosphorylation and respiratory electron transport were significantly enriched among downregulated genes, under exposure doses of 0.3 and 6.0 mg/kg (Figure 1H). Although oxidative phosphorylation was enriched for downregulated genes in both sexes, a larger number of associated genes were affected in male mice. Among these, *Cox6c* and *Sdhd* were identified as commonly downregulated in both males and females (Figure 1I,J). These findings highlight a dose- and sex-dependent disruption in hepatic energy metabolism following PFESA-BP2 exposure, marked by altered activity in key pathways such as the TCA cycle, β-oxidation, pyruvate metabolism, and oxidative phosphorylation.

### 3.3. PFOA and GenX Exposure Targets Fatty Acid and Lipid Metabolism

**Figure 2:**
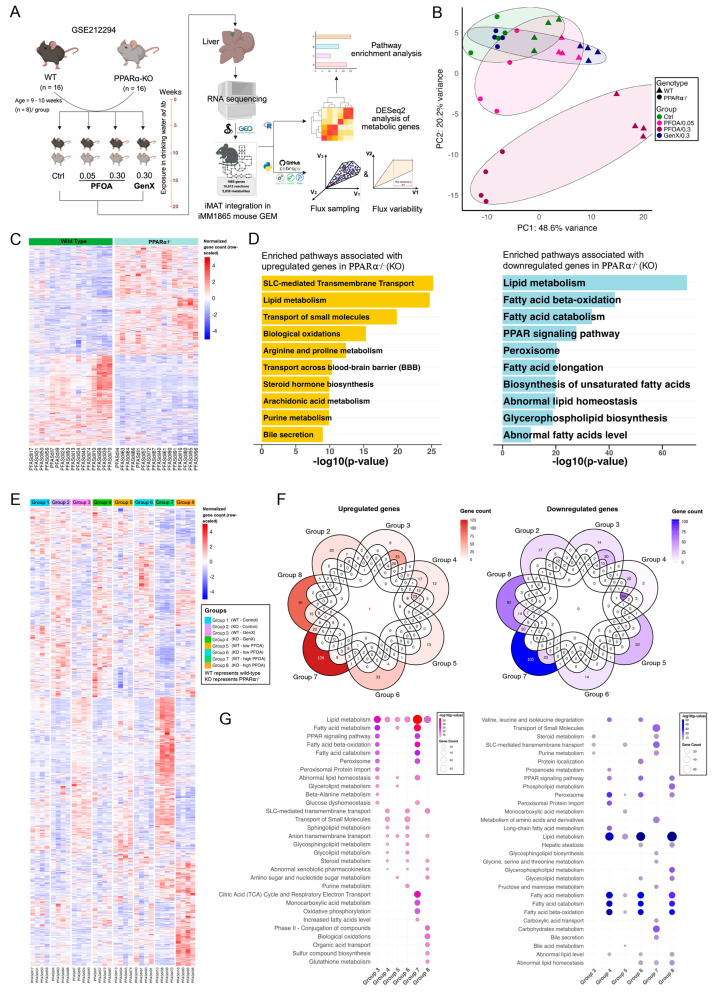
Hepatotoxicity of PFOA and GenX in WT and PPARα^−/−^ male mice. (**A**) Experimental design of the exposure conditions in GSE212294, with systems biology approaches applied in the present study. (**B**) Principal component analysis (PCA) of normalized gene count across WT and PPARα^−/−^ mice exposed to GenX and PFOA. (**C**) Heatmaps showing normalized and scaled gene count for differentially expressed genes (DEGs) in PPARα^−/−^ (KO; knockout) vs. wild-type mice (305 upregulated and 237 downregulated genes). (**D**) Top 10 enriched biological pathways associated with upregulated genes in PPARα^−/−^ mice (gold-colored) and downregulated genes (light blue-colored), where the *x*-axis represents the statistical significance of enrichment. (**E**) Heatmaps showing normalized and scaled gene count for DEGs across exposure groups vs. Group 1 (WT-control mice). (**F**) Venn diagrams of upregulated genes (gradient red-colored) and downregulated genes (gradient blue-colored) across exposure groups. (**G**) Top 30 enriched biological pathways associated with upregulated genes (gradient red-colored) and downregulated genes (gradient blue-colored) across exposure groups. Color intensity represents the statistical significance of enrichment (−log10 *p*-value), and the circle size reflects the number of genes associated with the corresponding pathway.

Long-chain PFAS such as PFOA (perfluorooctanoic acid) and short-chain PFAS such as GenX (hexafluoropropylene oxide dimer acid) are widely used PFAS with known environmental persistence and physiological effects. GenX was developed as a substitute for PFOA; however, both compounds have been reported to impact various physiological and molecular processes [11,73]. To further investigate PFAS-induced hepatotoxicity, with an emphasis on lipid metabolic alterations, we examined the impact of PFOA and GenX on hepatic transcriptome using mouse liver data from the GSE212294 study (Figure 2A) [21]. Principal component analysis (PCA) showed variances across exposure groups, with PC1 and PC2 explaining 48.6 and 20.2% of the total variance, respectively. The mice group exposed to high-dose PFOA (0.3 mg/kg) was distinctly clustered from other exposure groups. As the study involved wild-type and PPARα^−/−^ mice, PCA revealed a distinct separation between genotypes irrespective of the exposure (Figure 2B). DEG analysis of PPARα^−/−^ mice identified 305 upregulated and 237 downregulated metabolic genes (Figure 2C). Pathway enrichment analysis of upregulated genes in PPARα^−/−^ mice indicated solute carrier protein-mediated transport, lipid metabolism, biological oxidation, and amino acid metabolism in the top-enriched pathways. For the downregulated genes in PPARα^−/−^ mice, we identified lipid metabolism, FA oxidation, and FA metabolism in the top-enriched pathways (Figure 2D). PPARα is a well-established regulator of enzymes involved in lipid metabolism and FA oxidation; thus, its knockout has been consistently associated with impaired β-oxidation and the development of dyslipidemia [21,74].

We also investigated transcriptomic changes in response to GenX and PFOA exposure in both wild-type and PPARα^−/−^ mice. Transcriptomic profiling of GenX and PFOA exposure in wild-type and PPARα^−/−^ mice revealed significant differential metabolic gene expression across all groups. GenX exposure resulted in 103 upregulated and 75 downregulated genes in wild-type mice and 118 upregulated and 124 downregulated genes in PPARα^−/−^ mice. Low-dose PFOA induced 87/94 (up/down) DEGs in wild-type and 158/143 in PPARα^−/−^ mice. High-dose PFOA triggered the most extensive response, with 287/228 (up/down) DEGs in wild-type and 248/217 in PPARα^−/−^ mice (Figure 2E,F; Supplementary file S1). Enrichment analysis revealed that lipid metabolism was significantly enriched among upregulated genes in all exposure groups, with its most pronounced transcriptional changes observed in wild-type mice exposed to high-dose PFOA, followed by GenX (Figure 2G). This is concordant with the findings of Attema et al. [21], who showed that both GenX and high-dose PFOA cause lipid accumulation in the liver of wild-type mice. Moreover, high-dose PFOA and GenX exposure led to significant enrichment of fatty acid metabolism, β-oxidation, and PPAR signaling pathways among upregulated genes in wild-type mice. This effect was not observed in PPARα^−/−^ mice and low-dose PFOA exposure (Figure 2G). Some of the targeted genes included *Dgat1*, *Gpat4*, *Agpat2*, *Lpin2*, *Pgs1*, and *Ptdss2*, which are involved in glycerolipids and triglyceride biosynthesis. Other upregulated genes included *Lipe*, *Mgll*, *Pnpla2*, and *Abhd5*, which participate in lipolysis, and FA oxidation-associated genes such as *Acadvl*, *Acadm*, *Acads*, *Cpt2*, *Ehhadh*, *Hadha*, *Hadhb*, and *Slc25a20* (Supplementary file S1). Transcriptomic changes associated with energy metabolism, particularly oxidative phosphorylation and respiratory electron transport, were enriched only in WT mice exposed to high-dose PFOA. This indicates that elevated PFOA exposure may impair mitochondrial homeostasis and compromise energy production. Peroxisome proliferation was significantly enriched for upregulated genes following the exposure of GenX and high-dose PFOA in wild-type mice. However, this pathway was also found to be enriched for downregulated genes in PPARα^−/−^ mice across all exposures (Figure 2G; Supplementary file S1). Interestingly, lipid metabolism, fatty acid metabolism, and degradation, along with β-oxidation, were the notable enriched pathways among downregulated genes in PPARα^−/−^ mice at all exposures. The analysis suggests that both GenX and PFOA likely exert their lipid metabolic effects through PPARα activation, with a dose-dependent response particularly evident for PFOA.

### 3.4. Identifying Perturbations in Energy and Lipid Metabolism Due to PFAS Exposure Using Integrated Genome-Scale Metabolic Models

Considering the complexity of biological systems, we wanted to understand the mechanism by which PFAS exposure exerts perturbations on energy and lipid homeostasis. We used mouse genome-scale metabolic reconstruction, iMM1865 [44], as a template and expression data from GSE147331, GSE147332, and GSE212294 studies to generate context-specific GEM using the iMAT algorithm [58]. iMAT allows mapping the gene expression to their corresponding metabolic reactions, which can be tested for defining metabolic shifts under various conditions [58]. We obtained 20 context-specific GEMs of female BALB/c, 20 for male BALB/c, 18 for WT, and 18 for PPARα^−/−^, representing in silico models of all mice subjected to different exposure conditions. We performed flux balance analysis and flux variability analysis for these context-specific GEMs using COBRA Toolbox v3.0. We investigated the flux distribution for reactions that were part of energy and lipid metabolism. In this study, a subsystem is considered activated when the flux of its associated reactions is higher than that of the control groups (PFESA-BP2 study) or Group 1 (PFOA and GenX study) and suppressed when the flux is lower than the respective controls. Metabolic flux results from both FBA and FVA analyses are provided in Supplementary file S3.

**Figure 3:**
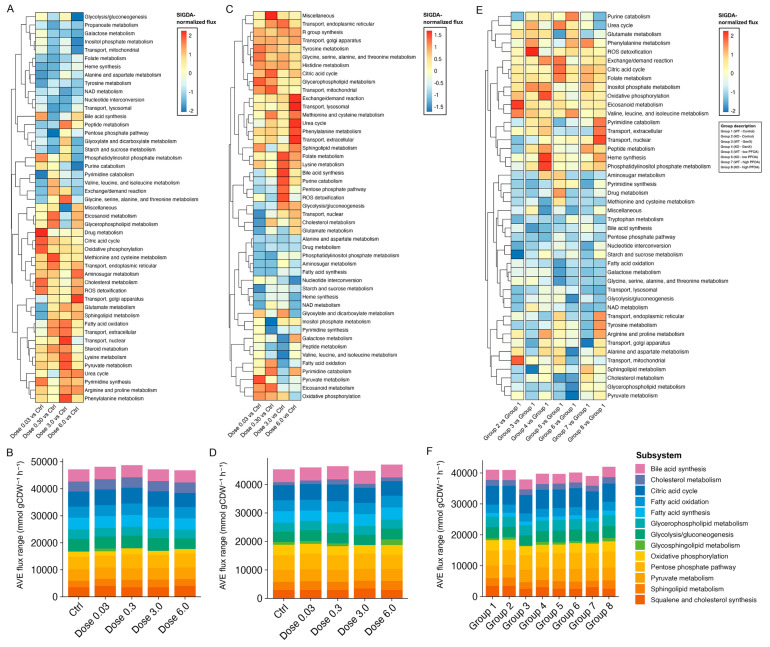
Flux states across exposure conditions in investigated mice. (**A**,**B**) Metabolic flux states of iMM1865 subsystems in context-specific GEMs, comparing with the control group in PFESA-BP2-exposed female BALB/c mice (GSE147332 study). (**C**,**D**) Metabolic flux states of male BALB/c GEMs, comparing with the control group under PFESA-BP2 exposure (GSE147331 study). (**E**,**F**) Metabolic flux states of mice exposed to PFOA and GenX (GSE212294 study). In (**B**,**D**,**F**), we show stacked bar plots, and the colors denote 13 selected model subsystems, associated with lipid and energy metabolism.

Under PFESA-BP2 exposure, male and female mice exhibited variation in the metabolic states. In female mice, lipid and energy metabolic subsystems were mostly enriched following the exposure compared with the control group, in a dose-dependent manner. Subsystems-level analysis identified the citric acid cycle, oxidative phosphorylation, fatty acid oxidation, and cholesterol metabolism, which were consistent with gene enrichment analyses. From our analysis, we identified phosphatidylinositol phosphate, amino sugar, and NAD metabolism, along with bile acid synthesis subsystems, suggesting additional metabolic alterations. Eicosanoid and glycerophospholipid metabolism were elevated at doses of 0.3 and 6.0 mg/kg, while suppressed at a 3.0 mg/kg dose. Sphingolipid metabolism was activated across all exposure doses, except for 0.03 mg/kg. In contrast, glycosphingolipid metabolism was significantly enriched only at the 0.3 mg/kg dose (Figure 3A,B). Notably, perturbations in metabolic fluxes of glycolysis/glycogenesis, mitochondrial transport, NAD metabolism, and folate metabolism suggest impaired mitochondrial function following exposure (Figure 3A).

In male mice, PFESA-BP2 exposure caused significant activation of the pentose phosphate pathway (PPP), purine metabolism, and glycolysis/glucogenesis at a dose of 3.0, followed by a dose of 6.0 mg/kg, while the TCA cycle was activated at all doses. The metabolic flux of oxidative phosphorylation was elevated in all doses, while suppressed at a dose of 6.0 mg/kg. Sphingolipid metabolism was activated in all doses but at a dose of 0.3 mg/kg, while glycerophospholipid metabolic flux was activated at all doses (Figure 3C). Glycosphingolipid metabolism was significantly enriched at a dose of 6.0 mg/kg, followed by other doses except 3.0 mg/kg (Figure 3D).

In PFOA and GenX case studies, subsystems associated with energy metabolism, such as the TCA cycle and oxidative phosphorylation, were activated, particularly in wild-type mice exposed to low-PFOA and GenX, respectively (Figure 3E). Fatty acid oxidation was consistently suppressed in all groups compared with the control (Figure 3E). Metabolic fluxes for reactions associated with cholesterol metabolism were significantly suppressed in mice exposed to low-PFOA while activated under high-PFOA exposure in wild-type mice. Glycerophospholipid metabolism was significantly suppressed in mice exposed to low-PFOA and GenX. Sphingolipid metabolism was significantly activated for high-PFOA while suppressed for GenX exposure in wild-type mice (Figure 3E,F). Pyruvate metabolism was significantly suppressed following low-PFOA exposure (Figure 3E), consistent with previous findings showing decreased levels of phosphoenolpyruvic acid in the liver of PFOA-exposed mice [75]. These findings highlight the value of in silico metabolic modeling in predicting perturbations in energy and lipid metabolism, offering potential metabolic signatures for assessing PFAS-induced hepatotoxicity.

### 3.5. PFESA-BP2 Exposure Causes Activation of Cholesterol Biosynthesis in a Dose-Dependent Manner

**Figure 4:**
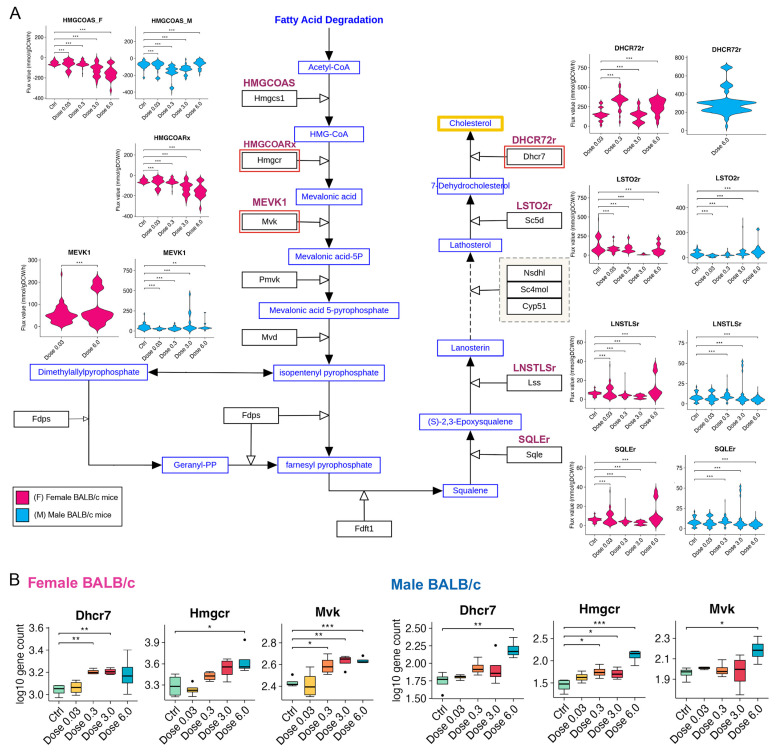
Flux distribution of metabolic reactions involved in cholesterol biosynthesis, investigated in female and male BALB/c mice under PFESA-BP2 exposure. (**A**) Metabolic fluxes of selected reactions, identified by their BiGG (Biochemical, Genetic and Genomic knowledgebase) IDs (red colored). The pathway schematic diagram was acquired from WikiPathways (WP103) database [77]. Positive flux values represent the forward direction of the reaction, while negative values indicate the reverse direction. (**B**) Normalized and log10 transformed expression values of selective genes, including *Hmgcr*, *Mvk*, and *Dhcr7*, associated with reactions HMGCOAS, MEVK1, and DHCR27r, respectively. Statistical analyses were performed using unpaired two-tailed *t*-tests, using Ctrl group as the respective comparison reference. A *p*-value < 0.05 was considered statistically significant (* *p* < 0.05, ** *p* < 0.01, *** *p* < 0.005).

Flux sampling enables a more comprehensive characterization of metabolic behavior beyond the optimal solution [76]. Since we captured lipid dysbiosis associated with PFESA-BP2 exposure, we wanted to investigate specific lipid species. Emerging evidence highlighted PFAS-related cholesterol perturbations, and we performed flux sampling to predict flux distribution through metabolic reactions involved in the terpenoid backbone and steroid biosynthesis, which contribute to cholesterol production (Figure 4A; Supplementary file S4). We examined the following reactions: HMGCOAS (catalyzed by Hydroxymethylglutaryl CoA synthase); HMGCOARx (Hydroxymethylglutaryl CoA reductase); MEVK1 (mevalonate kinase atp); SQLEr (Squalene epoxidase endoplasmic reticular NADP); LNSTLSr (lanosterol synthase); LSTO2r (lathosterol oxidase); and DHCR72r (7-dehydrocholesterol reductase).

According to our analysis, PFESA-BP2 exposure in both sexes led to activation of reactions in cholesterol biosynthesis, predicting cholesterol accumulation, particularly through elevating the metabolic fluxes of mevalonate and lanosterol biosynthesis (Figure 4A). Interestingly, sex-specific differences in fluxes were observed for key reactions such as HMGCOARx, MEVK1, and DHCR72r. These differences may be explained by the sex-dependent expression patterns of their corresponding enzymes (Figure 4B). The findings suggest that PFESA-BP2 exposure potentially causes cholesterol buildup through targeting mevalonate and lanosterol biosynthesis. However, such predictions need further investigation with experimental studies.

### 3.6. PFAS Exposure Causes Energy Dyshomeostasis via Targeting Carbon Metabolism and β-Oxidation

Lipids contribute to membrane integrity and are key regulators of energy homeostasis. Fatty acids act as key substrates in modulating the TCA cycle and β-oxidation [78]. Former reports highlighted the potential energy changes following PFAS exposure, where PFOA-exposed mice experienced it via AMPK/mTOR regulation [75]. PFOS-exposed mice had alterations in pyruvate metabolism and TCA cycle [10] while GenX-exposed mice exhibited changes in glucose and amino acid metabolism [12]. Since various energy-related pathways were enriched following PFAS exposure in the investigated studies, we wanted to spot potential energy targets of PFAS toxicity.

**Figure 5:**
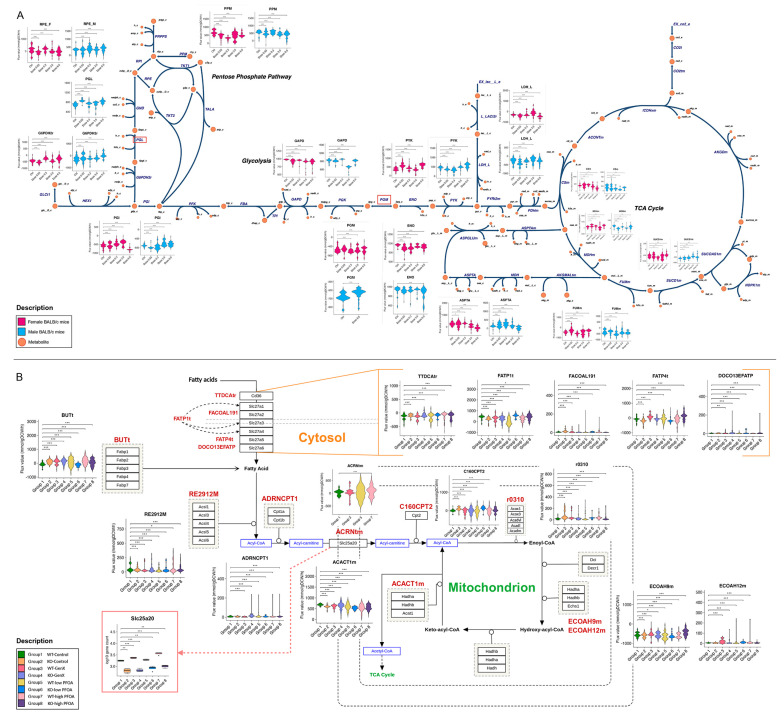
Flux distribution of metabolic reactions associated with energy metabolism across exposure conditions in investigated mice. (**A**) Reaction fluxes of pentose phosphate pathway (PPP), glycolysis, and citric cycle (TCA) in female and male BALB/c mice exposed to PFESA-BP2. Schematic diagram of the biological pathways was acquired from ESCHER database [79], where the orange-colored nodes represent metabolites, and reaction IDs are given to represent enzyme name catalyzing the reaction. (**B**) Reaction fluxes of fatty acid β-oxidation in mitochondria, including the transport reactions from extracellular and cytosol compartments in WT and PPARα^−/−^ male mice exposed to PFOA and GenX. The pathway schematic diagram was acquired from WikiPathways-(WP3588) database [78] with minor modifications, addressing the reactions of interest and related enzymes. The BIGG reaction IDs are given corresponding to their set of enzymes (highlighted in red color). Statistical analyses were performed using unpaired two-tailed *t*-tests, using Ctrl group (for panel A) and WT-control group (for panel B) as the respective comparison references. A *p*-value < 0.05 was considered statistically significant (* *p* < 0.05, ** *p* < 0.01, and *** *p* < 0.005). KO represents the PPARα^−/−^ mice. Low- and high-PFOA are PFOA doses of 0.05 and 0.3 mg/kg.bw/day, respectively.

Therefore, we investigated the flux distribution of reactions involved in central carbon metabolism for mice exposed to PFESA-BP2 (Figure 5A; Supplementary file S4), including the pentose phosphate pathway, glycolysis, and the citric acid (TCA) cycle. Metabolic fluxes showed that PFESA-BP2 exerted perturbations in all reactions, with a dose-dependent pattern. No sex-specific differences were captured except in the case of PGL (catalyzed by 6-phosphogluconolactonase) and PGM (phosphoglycerate mutase) (Figure 5A; Supplementary file S4), potentially driven by the expression pattern of their associated enzymes. Since the reactions plotted here are bidirectional, it is difficult to determine the exact directionality of metabolic shifts. However, the predicted flux distributions, together with enrichment analysis, highlight potential metabolic signatures that could guide experimental investigations into PFAS-associated energy dyshomeostasis.

In the PFOA- and GenX-exposed mice study, we investigated the metabolic fluxes of mitochondrial β-oxidation (Figure 5B; Supplementary file S4). The metabolic fluxes showed shifts with genotype- and dose-dependence. For instance, PPARα^−/−^ mice (Groups 2, 4, 6, and 8) often show lower flux through mitochondrial reactions ACACT1m (catalyzed by acetyl CoA C acetyltransferase) and ECOAH9m (Enoyl-CoA hydratase, 3-Hydroxy-2-methylbutyryl-CoA forming), highlighting a PPARα dependence. High-PFOA dose (Groups 7 and 8) showed slightly more metabolic activation of reactions ECOAH9m and BUTt (butyrate transport). Notably, metabolic fluxes of ACRNtm (carnitine/acylcarnitine translocase) were captured exclusively in wild-type mice across all exposures. This aligns with its significantly suppressed gene expression following PPARα knockout, suggesting that the loss of PPARα impairs carnitine transport into mitochondria and may underlie the observed suppression of β-oxidation in PPARα^−/−^ mice [21,74] (Figure 5B; Supplementary file S4).

## 4. Discussion

We carried out a systematic study to identify metabolic perturbations in mice in response to PFAS exposure. We analyzed liver transcriptome data of mice exposed to long- and short-chain PFAS. The main findings of our study are as follows: (1) PFESA-BP2 exposure caused hepatotoxicity in mice through targeting lipid and energy metabolism via activating SREPF and PPAR signaling. The metabolic perturbations included upregulation in fatty acid metabolism, cholesterol biosynthesis, and β-oxidation while downregulating the metabolism of triglycerides and glycerophospholipids. Such changes were dose- and sex-dependent. A broader systemic changes targeted the blood–brain barrier, causing potential neurotoxicity; (2) flux simulation of metabolic reactions in PFESA-BP2 exposed mice showed further perturbations targeting energy metabolism such as TCA, oxidative phosphorylation, and pyruvate metabolism while potentially activating cholesterol biosynthesis via targeting mevalonate and lanosterol synthesis; (3) PFOA and GenX exposure caused perturbations in lipid and energy homeostasis via activating PPARα; and (4) computational predictions elaborated the potential disturbances in mitochondrial β-oxidation via targeting the carnitine shuttle, following PFOA and GenX exposure in a PPARα-dependent manner.

Using transcriptomic data from Lang et al. [30], we found that PFESA-BP2 induces sex- and dose-dependent hepatic lipid dyshomeostasis, presumably regulated by SREBF and PPAR signaling (Figure 1G,J). These findings align with prior studies reporting altered serum cholesterol [80] and triglyceride levels [81] following PFAS exposure. Moreover, PFESA-BP2 induced dose-specific hepatic energy shifts—particularly in TCA and pyruvate metabolism, aligning with prior PFAS studies linking carbon metabolic disruption to TCA, glycolysis, and butanoate pathways [11,82], suggesting mitochondrial dysfunction and consistent with reported PFAS-related mitochondrial impairments [83]. The liver represents the hub of metabolism, and former research implied the influence of its metabolic dysfunction on neuronal complications and cognition [84]. Downregulation of blood–brain barrier transport genes (SLCs, *Fabp5*, and *Abcc9*) under high-dose exposure in female mice, suggests liver–brain axis disruption, supported by FABP5’s known role in docosahexaenoic acid (DHA) uptake and cognitive function [85]. This effect was not observed in male mice, highlighting sex-specific responses to PFAS exposure and underscoring the importance of considering sex as a biological variable in PFAS toxicity studies. Collectively, our findings highlight the extensive impact of PFESA-BP2 on hepatic lipid and energy homeostasis, suggesting systemic toxic effects that may extend to the brain and potentially impair cognitive function.

High-dose PFOA induced strong hepatic lipid metabolic responses in wild-type mice—including β-oxidation, lipolysis, and triglyceride biosynthesis—which were abolished in PPARα^−/−^ mice, underscoring PPARα’s regulatory role in PFAS toxicity [21]. Our findings are consistent with earlier studies highlighting the positive correlation between serum triglycerides and PFOA exposure [86,87,88]. High-dose PFOA enriched oxidative phosphorylation and respiratory electron transport in wild-type mice, indicating mitochondrial disruption (Figure 2G; Supplementary file S1), consistent with previous reports of PFOA-induced mitochondrial abnormalities [5]. GenX and high-dose PFOA enriched peroxisome proliferation and lipid metabolic pathways in wild-type mice, while PPARα^−/−^ mice showed consistent downregulation across all doses (Figure 2G; Supplementary file S1). Additionally, peroxisome proliferation was enriched only in wild-type mice, consistent with classical PPARα activation effects [74]. Our results provide transcriptional evidence, conforming with previous reports of lipid accumulation and mitochondrial abnormalities induced by PFAS [5,75].

While transcriptomic analysis offered valuable insights into the molecular pathways affected by PFAS congeners, the underlying mechanisms driving such toxicity remain incompletely understood. To overcome this limitation, we integrated transcriptomic data with genome-scale metabolic modeling to predict functional metabolic shifts and uncover system-level disruptions beyond gene expression alone. Computational predictions revealed dose- and sex-specific PFESA-BP2-induced shifts, activating TCA cycle, oxidative phosphorylation, FA oxidation, cholesterol, and complex lipid metabolism pathways (Figure 3A–D). Notably, glycerophospholipid activation aligns with lipidomic data showing elevated hepatic phosphatidylglycerols and phosphatidylinositols in mice after PFESA-BP2 exposure [8]. Additionally, the same analysis highlighted the potential influence of PFOA and GenX exposure on metabolic fluxes of reactions associated with energy metabolism and lipid pathways through a PPARα-independent manner (Figure 3E,F). Such predictions further align with the findings from a systematic review including cross-sectional studies on human metabolome and PFAS exposure [82]. Their observations showed significant metabolic changes, including glycerophospholipids, sphingolipids, steroids and steroid derivatives, and carboxylic acids, in association with exposure to various PFAS congeners [82]. In their investigated PFAS congeners [82], PFOA was amongst the long-chain PFAS, which upregulated human metabolome of fatty acids [89,90,91], acylcarnitines [89,91,92], phosphosphingolipids [91,93], and glycerophosphoinositols [91].

While FBA and FVA provide insights into flux distributions, they focus on optimizing a single objective function under the constraints of the genome-scale model (GEM). However, to capture the full range of feasible metabolic states of individual reactions while satisfying mass balance and flux bounds, we performed flux sampling. Metabolic flux simulations through flux sampling revealed PFESA-BP2-induced activation of cholesterol biosynthesis via increased flux through HMGCOAS, HMGCOARx, and MEVK1, with sex-specific differences in DHCR72r, suggesting cholesterol buildup through the mevalonate–lanosterol pathway (Figure 4). While this aligns with our enrichment results, it contrasts with Conley et al. [4], who observed cholesterol reduction at a much higher dose (30 mg/kg). Flux simulations are concordant with published epidemiological reports indicating PFAS exposure increases lipids such as cholesterol in human blood samples [94]. This observation is also supported by cross-sectional studies demonstrating a positive correlation between PFESA-BP2 exposure and elevated high-density lipoprotein (HDL) cholesterol levels in human blood [95]. Further simulations showed PFESA-BP2 disrupted carbon metabolism in a dose-dependent manner, targeting glycolysis, the TCA cycle, and PPP via G6PDH2r, PGM, and MDHm (Figure 5A). These findings support our hypothesis that lipid dyshomeostasis may extend to energy imbalance, offering new metabolic markers for future studies. Modeling simulations depicted both PFOA and GenX to cause genotype- and dose-dependent changes in mitochondrial β-oxidation disruption, with reduced ACACT1m and ECOAH9m in PPARα^−/−^ mice and exclusive ACRNtm activation in wild-type mice, indicating impaired mitochondrial fatty acid transport upon PPARα loss (Figure 5B). Previous studies have associated PFAS exposure with acylcarnitines (ACs) upregulation and enhanced β-oxidation [96,97], which is consistent with our computational results of GenX- and PFOA-exposed wild-type mice. Our in silico flux analysis results are also concordant with cross-sectional studies showing the elevated levels of acylcarnitines in the human metabolome associated with PFAS exposure [89,91,92]. Elevation in acylcarnitines implies an incomplete processing of long-chain fatty acids through β-oxidation and a limited availability of intermediates required for TCA modulation [98]. Altogether, this study reveals that PFAS congeners, particularly PFESA-BP2, PFOA, and GenX, induce sex- and dose-dependent disruptions in hepatic energy and lipid metabolism. Through integrative transcriptomics and metabolic modeling, we identified critical metabolic disruptions that offer mechanistic insights and reveal potential biomarkers of PFAS-induced toxicity.

### 4.1. Limitations of the Study

Our present work has certain limitations. First, our analysis primarily focused on metabolic genes, representing only a subset of the liver transcriptome, while excluding signaling genes and other protein-coding elements that potentially play crucial roles in regulating signaling cascades that influence downstream metabolic alterations following PFAS exposure. Second, our study relied on the integration of transcriptome data with genome-scale metabolic models (GEMs) to predict changes in metabolic fluxes. In the absence of matched metabolomics data, we were unable to validate our predictions. Metabolomics complements transcriptomics by capturing downstream biochemical changes, and when integrated, the two provide a systems-level perspective on molecular and phenotypic responses to environmental toxicants [99]. Future work should focus on generating transcriptome and metabolomics data from matched samples for validation purposes and providing a holistic view of the metabolic perturbations in the system due to PFAS exposure.

### 4.2. Applications of This Study

Our systematic study explored the molecular changes associated with three PFAS congeners, PFESA-BP2, PFOA, and GenX, using transcriptomic and systems biology approaches to identify liver metabolic shifts in two murine models. The analyses identified important metabolic signatures associated with hepatic lipid and energy dyshomeostasis. The enrichment analysis of PFESA-BP2-exposed mice revealed potential disruption of the blood–brain barrier via downregulation of critical transport-related genes, raising concerns about systemic neurotoxicity. Computational simulations further predicted cholesterol accumulation and carbon metabolism perturbations in response to PFESA-BP2. Additionally, flux simulations identified potential signatures for GenX- and PFOA-induced hepatotoxicity, particularly involving impaired mitochondrial β-oxidation through suppressed carnitine transport in a PPARα-dependent manner. These findings highlight key molecular pathways and candidate targets that warrant further experimental validation to better understand PFAS-associated toxicity.

## Data Availability

Transcriptomic data with the accession numbers GSE147331, GSE147332, and GSE212294 were downloaded from the GEO database. The normalized gene expression mapped to metabolic genes in iMM1865, the mouse genome-scale metabolic model, along with all analysis scripts, are publicly available on GitHub at (https://github.com/BaloniLab/PFAS_Congeners_Mouse_GEMs.git) (1 June 2025).

## Supplementary Materials

The following supporting information can be downloaded at https://www.mdpi.com/article/10.3390/metabo15080499/s1, Supplementary file S1: Results of differentially expressed genes and enrichment analysis; Supplementary file S2: PCA plot of PFESA-BP2 exposed female and male mice; Supplementary file S3: List of files for FBA and FVA analyses of mouse GEMs; Supplementary file S4: List of reactions presented in Figure 4 and Figure 5.

## Abbreviations

The following abbreviations are used in this manuscript:

AMPK	AMP-activated protein kinase pathway
ACs	Acylcarnitines
BBB	Blood–brain barrier
BiGG	Biochemical, Genetic, and Genomic Knowledgebase
COBRA	Constraint-based reconstruction and analysis
DEGs	Differentially expressed genes
FA	Fatty acid
FBA	Flux balance analysis
FVA	Flux variability analysis
GEM	Genome-scale metabolic model
GenX	Hexafluoropropylene oxide dimer acid
GEO	Gene expression omnibus
GSEA	Gene set enrichment analysis
iMAT	Integrative metabolic analysis tool
mTOR	Mammalian target of rapamycin pathway
PPP	Pentose phosphate pathway
PPAR	Peroxisome proliferator-activated receptor
PFAS	Per-(poly) fluoroalkyl substances
PFOA	Perfluorooctanoic acid
PFESA-BP2	7H-Perfluoro-4-methyl-3,6-dioxaoctanesulfonic acid
SREPFs	Sterol regulatory element-binding transcription factors
TCA	Tricarboxylic acid
