# Supplementary figures and images for: Investigating Lipid and Energy Dyshomeostasis Induced by Per- and Polyfluoroalkyl Substances (PFAS) Congeners in Mouse Model Using Systems Biology Approaches
Source: Metabolites. 2025 Jul 24;15(8):499. doi: 10.3390/metabo15080499 (PMC12388551; doi:10.3390/metabo15080499)

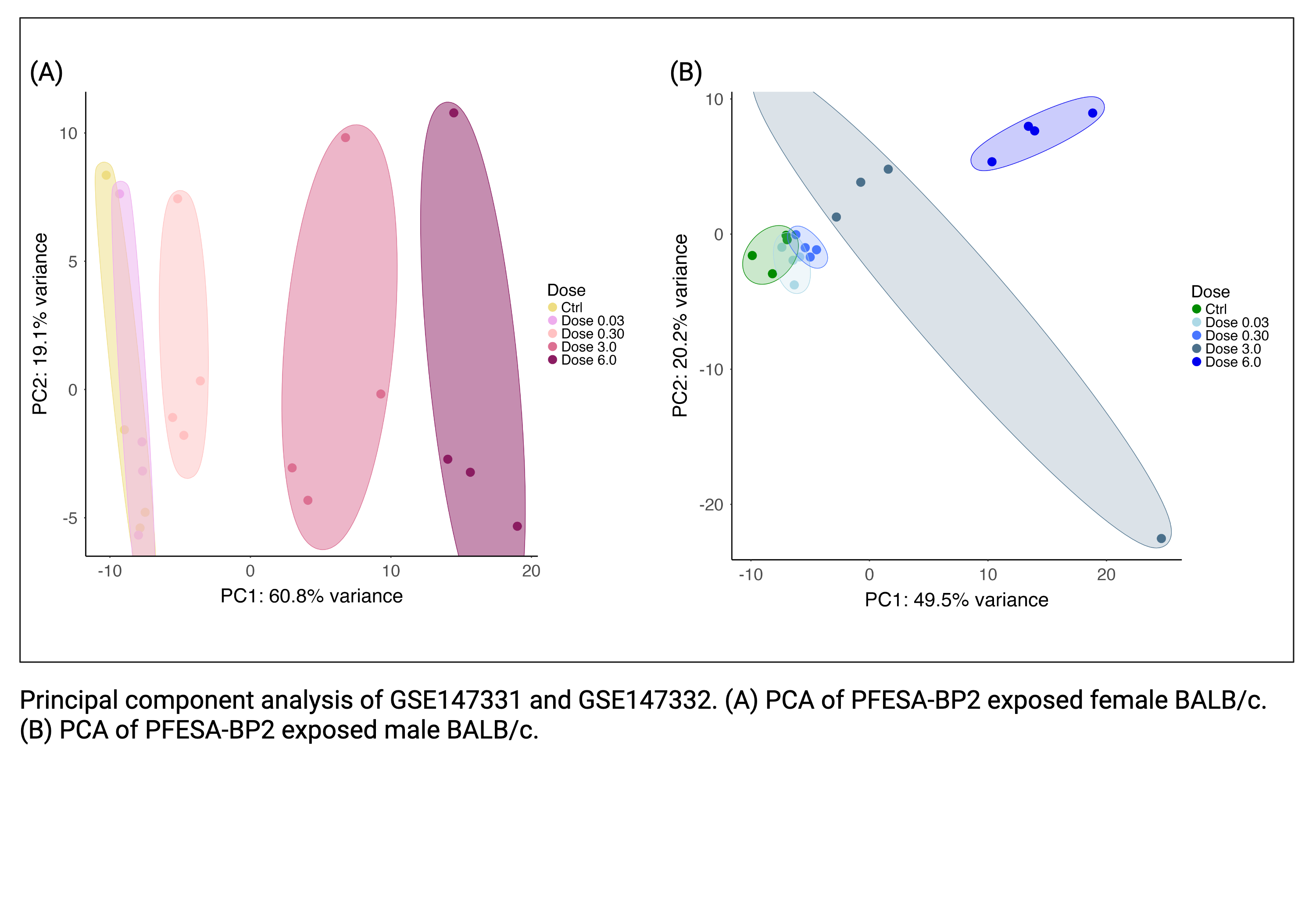

Supplement: Supplementary file 1 [file metabolites-15-00499-s001.zip › Supplementary file S2.jpeg]
